# Supplementary material for: Perfusion Techniques in Kidney Allograft Preservation to Reduce Ischemic Reperfusion Injury: A Systematic Review and Meta-Analysis
Source: Antioxidants (Basel). 2024 May 24;13(6):642. doi: 10.3390/antiox13060642 (PMC11200710; doi:10.3390/antiox13060642)
Supplement: Supplementary file 1 [file antioxidants-13-00642-s001.zip › antioxidants-2959729-supplementary.pdf]

SUPPLEMENTARY MATERIALS:

1. **Table S1.** Summary of search terms used.
2. **Table S2.** Summary of MP vs. SCS studies.
3. **Table S3.** Summary of NRP vs. ISP studies.

Table S1. Summary of search terms used.

Database	Search Strategy	Total Articles in Search
PubMed	((Cold storage OR Static storage OR Preservation solutions OR machine perfusion OR ex vivo OR hypothermic machine perfusion OR normothermic machine perfusion OR subnormothermic machine perfusion OR controlled oxygenated rewarming OR organ perfusate OR Histidine tryptophan OR Wisconsin Solution OR Organ Preservation*[MeSH] OR Perfusion*[MeSH] OR Hypothermia*[MeSH] OR Refrigeration*[MeSH]) AND ("Chronic Kidney Disease" OR "End stage Kidney Disease" OR "End stage Renal Disease" OR "ESRD" OR "ESKD" OR Kidney Transplant* OR Renal Transplant* OR Deceased Cardiac Donor OR Deceased Brain Death Donor OR Extended Criteria Donor OR Marginal Graft OR Kidney Diseases[MeSH] OR Kidney Transplantation*[MeSH] OR "Kidney/surgery"[MAJR] OR Tissue Donors [MeSH] OR Humans[MeSH]) AND (Oxidative stress OR Inflammation OR Mortality OR delayed graft function OR primary nonfunction OR ischemia OR reperfusion OR injury OR graft survival OR graft rejection OR re-transplantation OR Delayed Graft Function[MeSH] OR Graft Survival[MeSH] OR Graft Rejection[MeSH] OR "Kidney Transplantation/mortality"[MeSH] OR Nephrectomy[MeSH] OR Reperfusion Injury[MeSH]))	4251
Embase	(Cold storage OR Static storage OR Preservation solutions OR machine perfusion OR ex vivo OR hypothermic machine perfusion OR normothermic machine perfusion OR subnormothermic machine perfusion OR controlled oxygenated rewarming OR organ perfusate OR Histidine tryptophan OR Wisconsin Solution OR Organ Preservation* OR Perfusion* OR Hypothermia* OR Refrigeration*)	800
	("Chronic Kidney Disease" OR "End stage Kidney Disease" OR "End stage Renal Disease" OR "ESRD" OR "ESKD" OR Kidney Transplant* OR Renal Transplant* OR Deceased Cardiac Donor OR Deceased Brain Death Donor OR Extended Criteria Donor OR Marginal Graft OR Kidney Diseases OR Kidney Transplantation* OR Tissue Donors OR Humans)	
	(Oxidative stress OR Inflammation OR Mortality OR delayed graft function OR primary nonfunction OR ischemia OR reperfusion OR injury OR graft survival OR graft rejection OR re-transplantation OR Delayed Graft Function OR Graft Survival OR Graft Rejection OR Mortality OR Nephrectomy OR Reperfusion Injury)	

Cochrane	(Cold storage OR Static storage OR Preservation solutions OR machine perfusion OR ex vivo OR hypothermic machine perfusion OR normothermic machine perfusion OR subnormothermic machine perfusion OR controlled oxygenated rewarming OR organ perfusate OR Histidine tryptophan OR Wisconsin Solution OR Organ Preservation* OR Perfusion* OR Hypothermia* OR Refrigeration*) AND (“Chronic Kidney Disease” OR “End stage Kidney Disease” OR “End stage Renal Disease” OR “ESRD” OR “ESKD” OR Kidney Transplant* OR Renal Transplant* OR Deceased Cardiac Donor OR Deceased Brain Death Donor OR Extended Criteria Donor OR Marginal Graft OR Kidney Diseases OR Kidney Transplantation* OR Tissue Donors OR Humans) AND (Oxidative stress OR Inflammation OR Mortality OR delayed graft function OR primary nonfunction OR ischemia OR reperfusion OR injury OR graft survival OR graft rejection OR re-transplantation OR Delayed Graft Function OR Graft Survival OR Graft Rejection OR Mortality OR Nephrectomy OR Reperfusion Injury)	6609
Scopus	"Cold storage" OR "Static storage" OR "Preservation solutions" OR "machine perfusion" OR "hypothermic machine perfusion" OR "controlled oxygenated rewarming" OR "organ perfusate" OR "Histidine tryptophan" OR "Wisconsin Solution" OR Organ Preservation* OR Perfusion* OR Hypothermia* OR Refrigeration*	
	“Chronic Kidney Disease” OR “End stage Kidney Disease” OR “ESRD” OR Kidney Transplant* OR Renal Transplant* OR "Deceased Cardiac Donor" OR "Deceased Brain Death Donor" OR "Extended Criteria Donor" OR "Marginal Graft" OR "Kidney Diseases" OR Kidney Transplantation* OR "Tissue Donors" OR Humans	
	"Oxidative stress" OR Inflammation OR Mortality OR "delayed graft function" OR ischemia OR reperfusion OR injury OR "graft survival" OR "graft rejection" OR "re-transplantation" OR "Delayed Graft Function" OR "Graft Survival" OR "Graft Rejection" OR Mortality OR Nephrectomy OR Reperfusion Injury	2108
Manual Selection		13
Total		13781

Table S2. Summary of MP vs. SCS studies.

Authors	Year	Study Design	Centers, Country	HMP Device	SCS Solution	Donor Types Involved	Main Findings
Alijani et al. [1]	1985	RCT, kidney pairs	Multicenter, USA	Waters MOX-100	Euro-Collins	DBD	Decreased incidence of post-transplantation dialysis (17% vs 63%, P<0.01) and superior early graft function among MP grafts. Higher dialysis requirements among SCS grafts offsets cost savings achieved through transporting cold stored allografts.
Merion et al. [2]	1990	Prospective, kidney pairs	Multicenter, USA	Waters MOX-100	Euro-Collins	DBD	Recommended SCS over HMP due to cost savings and lack of observed superior outcomes among the HMP cohort. There were no differences in graft function, dialysis requirement, ischemia time, and graft loss within 30 days of follow-up.
Matsuno et al. [3]	1994	Prospective, kidney pairs	Single center, Japan	APS-02 Nikiso	UW, Euro-Collins	DCD	MP associated with higher rates of immediate function (38.5% vs. 7.6%), and lower rates (61.5% vs. 84.6%) and duration (8.0 days vs. 12.4 days) of DGF despite history of longer CIT (11.9 hours vs. 6.08 hours, P<0.05)
Daemen et al. [4]	1997	Retrospective	Multicenter, Europe	N/A	UW	DCD for MP, DBD for CS	MP were applied to DCD grafts while SCS were applied to DBD grafts. No difference in posttransplant diuresis, duration of DGF, number of dialyses, or rejection episodes within 3 months. PNF (19% vs. 7%) rates were higher in MP group. The study concluded that, the objective of MP in DCDs were not to repair grafts, but rather to minimize additional damage. Because DCD grafts already suffer more ischemic damage, further insults that threaten graft function, like SCS, prolonged preservation and cyclosporine nephrotoxicity, should be avoided so as to obtain optimal transplant results.
Kosieradzki et al. [5]	1999	Prospective, kidney pairs	Single center, Poland	Waters MOX-100	UW	DCD	There were reduced requirements for posttransplant dialysis (1.54 vs. 2.8, P<0.05) among the HMP cohort, despite longer total ischemia times (33 hours 41 minutes vs. 27

							hours 28 minutes, P<0.001). There were no differences in acute or chronic rejection episodes. Patients in the HMP group had improved graft function as measured by serum creatinine at 12- (P<0.05) and 24-months (P<0.01) of follow-up.
Sellers et al [6].	2000	Prospective	Single center, USA	Waters MOX-100	UW	DBD	Graft survival was not significantly different between groups, despite worse donor (higher donor age, higher donor creatinine, P<0.001) in the MP group. Survival of MP kidneys were not affected by longer implantation WIT, nor total ischemic time. DGF was more likely after CS preservation (20.2% versus 8.8%, p = 0.001).
Kwiatkowski et al. [7]	2007	Prospective	Single center, Poland	Waters MOX-100	MPS-2	DBD, DCD	No difference in DGF, PNF, or overall mortality. 5-year graft survival was better in MP-stored than in CS-stored kidneys (68.2% vs. 54.2%, P=0.02), despite longer cold ischemia time (33.7 hours vs. 27.5 hours, P<0.001) and higher donor serum creatinine (1.76 m/dL vs. 1.41 mg/dL, P=0.003) in the MP group. Also at 5 years post-transplantation, recipients of HMP grafts had less patients who returned to dialysis (20% vs. 36%, P=0.01). HMP decreased the odds of a negative outcome by 53% on logistic regression analysis.
Plata-Munoz et al. [8]	2008	Prospective	Single center, UK	LifePort	Marshall's	cDCD, ECD	Among cDCD grafts, HMP resulted in lower rates of DGF (53.3% vs. 86.6% P<0.001) and lengths of hospitalization (10 vs. 14 days P<0.033) in the cDCD-PP group. Short-term graft function (7, 30 days and 6 and 12 months) as measured by serum creatinine, were also improved in the HMP cohort (P<0.05). No difference in acute rejection, PNF, and postoperative and 1-year graft survival.
Reznik et al. [9]	2008	Prospective, kidney pairs	Single center, Russia	LifePort	N/A	uDCD	Among uDCD grafts, HMP cohorts had reduced rates of DGF (52% vs 82%, P<0.001), mean dialysis procedures within 30 postoperative days (2.3 vs. 4.9, P<0.05), length of hospital stay (29.3 vs. 43.8 days, P<0.001). Graft functioning, as measured by serum creatinine, were superior

							at post-operative day 1, 21, and 90. Perfusion parameters prognosticated graft functioning: grafts that functioned immediately had resistive indexes that normalized within 1-3 hours while those with DGF took up to 12 hours. Chemically damaged kidneys are suitable, but should have a recovery pump perfusion period of at least 10 hours.
Shah et al. [10]	2008	Retrospective, kidney pairs	Single center, USA	Waters MOX-100	UW, HTK/Celsior	SCD, ECD	The incidence of DGF (5% vs. 35%, P<0.01) and posttransplant dialysis (5% vs. 30%, P<0.01) was reduced in the HMP cohort. There were no differences in 1-year graft and patient survival.
Kwiatkowski et al. [11]	2009	RCT, kidney pairs	Single center, Poland	Waters MOX-100	MPS-2	DBD, DCD	Kidney storage by HMP reduced the number of patients who return to dialysis treatment at 10 years of follow-up (50% vs. 25%, p=0.02). There were no differences in donor and recipient characteristics, or graft survival between groups.
Jochmans et al. [12]	2010	RCT, kidney pairs	Multicenter, Europe	LifePort	UW, HTK/Celsior	cDCD	HMP reduced the incidence of DGF (53.7% vs. 69.5%, P=0.007) and the odds of DGF after adjusting for clinicodemographic covariates (aOR: 0.43; 95%CI 0.20–0.89; P=0.025). Other risk factors associated with reduced odds of DGF were donor age (OR 1.04, 95%CI 1.01-1.08), recipient age (OR 1.04, 95%CI 1.00-1.08), CIT (OR 1.01, 95%CI 1.01-1.21), and WIT (OR 3.40, 95%CI 1.87-6.17). HMP grafts had a higher creatinine clearance up to 1 month after transplantation (P = 0.027). One-year graft and patient survival was similar.
Watson et al. [13]	2010	RCT, kidney pairs	Multicenter, UK	LifePort	UW	cDCD	There was no difference in the incidence of DGF, renal function at 3 and 12 months, graft and patient survival. No HMP advantage was observed in cDCD grafts (with mean cold ischemic times around 14 hours).
Abboud et al. [14]	2011	Prospective, kidney pairs	Single center, France	LifePort	UW	ECD	The rate of DGF was lower (9% vs. 31.8%, p=0.021) in the PPP group. Donor and recipient clinicodemographic covariates were similar in both groups besides that those preserved by HMP had a higher number of HLA-A, B, and DR

							mismatched antigens with the recipient than the SCS cohort (4.0 vs. 3.1, P=0.012). Graft function, survival, and overall patient survival were similar at 1, 3, and 12 months of follow-up.
Treckman et al. [15]	2011	RCT, kidney pairs	Multicenter, Europe	LifePort	UW	ECD	HMP was associated with a 56% and 65% reduction in odds of DGF (OR 0.46, 95%CI 0.21-0.99) and 1-year graft survival (HR 0.35, 95%CI 0.15-0.86). The incidence of PNF was lower in the HMP group (3% vs. 12%, P=0.04). Mean creatinine clearance at 1-year follow-up was higher in HMP (78 ml/min vs. 69 ml/min, P=0.01). No difference in duration of DGF, CNI toxicity, and acute graft rejection. However, the incidence of DGF markedly reduced the graft survival in both HMP and SCS groups.
Gallinat et al. [16]	2012	RCT, kidney pairs	Multicenter, Europe	LifePort	UW	ECD	Only CIT was an independent risk factor for the development of DGF (OR 1.2, P <0.001). PNF was reduced in the HMP group (3.5% HMP versus 12.9% CS, P = 0.02). The 1-year graft survival rate was significantly improved after MP in recipients who developed DGF (84% vs. 48%, P=0.01), though there was no difference in overall 1-year patient and graft survival . There was also no difference in rates of DGF.
Hanf et al. [17]	2012	Prospective	Single center, France	RM3	IGL-1	uDCD, ECD	Kidneys from ECD were preserved by SCS while those from uDCD were preserved by HMP. There was no difference in long-term graft function or histological assessments of interstitial lesions, although patients in the uDCD cohort had higher rates of DGF (81.5% vs. 27.6%, P<0.001), mean number of dialysis sessions (4.7 vs. 0.7, P<0.001), dialysis days (15.6 vs. 2.8 days, P<0.001), and days until graft function recovered (17.8 vs. 5.0 days, P<0.001). There was no difference in acute rejection or PNF rates.
Moers et al. [18]	2012	RCT, kidney pairs	Multicenter, Europe	LifePort	UW	DBD, cDCD	HMP reduced the odds of DGF after adjusting for clinicodemographic covariates (aOR: 0.57; 95%CI 0.36-0.88; P=0.01). Other risk factors associated with reduced odds of DGF were

							donor age (OR 1.03, 95%CI 1.00-1.06), CIT (OR 1.08, 95%CI 1.03-1.14), duration of pretransplant dialysis (OR 1.16, 95%CI 1.03-1.31), repeat transplant recipient (OR 3.01, 95%CI 1.5-5.18), and DCD status (OR 17.2, 95%CI 8.16-36.2). HMP was also reduced the odds of 1-year graft failure (OR 0.52, 95%CI 0.29-0.93), as well as recipient age (HR 0.97, 95%CI 0.95-1.00), donor age (HR 1.05, 95%CI 1.01-1.10). Within the first year of transplantation, a serious adverse event occurred in 23% HMP vs 26% SCS, while a minor event occurred in 51% HMP vs. 44% SCS.
Cannon et al. [19]	2013	Retrospective	Multicenter, USA	Various	Various	DBD, DCD, ECD, SCD	Rates of DGF were similar in the overall cohort, though they are reduced (21.1% vs. 29.1% , P<0.001) after propensity match and analyzing kidney pairs (19.7% vs. 27.5%, P<0.001). No difference in the hazard for graft failure in the propensity matched or kidney pair analysis. The lack of difference in DGF in the overall cohort are likely due to the preferential use of HMP for marginal kidneys.
Sedigh et al. [20]	2013	Retrospective	Single center, Sweden	LifePort	UW, HTK/Celsior	ECD, SCD	There were no differences in DGF, PNF, acute rejection, or overall graft survival between HMP and SCS. However, grafts that underwent HMP had higher terminal serum creatinine levels (0.83 mg/dL vs. 0.74 mg/dL, P=0.43). When using SCD grafts only, HMP had a lower incidence of DGF (0% vs. 21.4%, P=0.046)
Tozzi et al. [21]	2013	Prospective	Single center, Italy	RM3	HTK/Celsior, Genzyme Sanofi	DCD	There were no differences in DGF or graft function. However, HMP had a lower profile of early inflammatory biomarkers: TNF-α, IL-2, IL-1β, and sICAM-1.
Dion et al. [22]	2015	Retrospective, kidney pairs	Single center, Canada	LifePort	N/A	DBD, DCD, ECD, SCD	Despite longer cold ischemia times (18.8 vs. 8.8 hours, P=0.001), grafts maintained with HMP had lower resistive indices (P=0.005) and higher eGFR at post-transplant follow-up. The effect was greatest among grafts obtained from DCD,

							which can be appreciated for as long as 2 years after transplantation (P=0.008).
Guy et al. [23]	2015	Prospective	Single center, UK	LifePort	KPS-1	DBD, DCD	Kidney transplanted from 8am-8pm were maintained in SCS, while those after hours were maintained in HMP. Rates of DCD (12% vs. 26%, P=0.035) and DGF (27% vs. 47%, P=0.012) were lower in the HMP group despite longer CIT (23.9 vs. 13 hours, P<0.001). There was no difference in PNF, hospital length of stay, post-transplant complications, patient or graft survival. Among HMP grafts, there was a large decline in resistance during the first hour of HMP, though there were no significant differences in resistance between grafts with immediate function or DGF.
Yao et al. [24]	2015	Prospective, kidney pairs	Single center, China	LifePort	KPS-1	DCD	HMP group had lower rates DGF (2.6% vs. 17.6%, P=0.029) and higher graft functioning at 6-month follow-up (eGFR 100.8 vs. 85.2, P=0.013). No difference in postoperative rejection incidence between the 2 groups.
Forde et al. [25]	2016	Retrospective	Single center, Ireland	LifePort	UW	ECD	Grafts maintained by HMP were from older donors (58.4 vs. 55.7 years, P<0.001) and had lower CIT (15.6 vs. 17.9 hours, P<0.001). HMP had improved graft function as measured by mean serum creatinine; 1.64 vs 1.84 mg/dL, P=0.010 at 1 month; 1.56 vs 1.75 mg/dL, P=023 at 3 months. There were no differences in DGF, graft survival and function at 1-year follow-up
Moser et al. [26]	2017	Retrospective	Single center, Canada	LifePort	KPS-1	DBD, DCD	Grafts maintained by HMP had higher CrCl from days 2 through 7, and 1 year post-transplant despite longer CIT and WIT. The vascular resistance (measured by RI) in the HMP group were lower. There was no difference in acute rejection within 1 year of transplant.
Tedesco-Silva et al. [27]	2017	RCT, kidney pairs	Multicenter, Brazil	LifePort	SPS1, HTK/Celsior	DBD, ECD, SCD	HMP had lower incidence of DGF (61% vs. 45%, P=0.031) and adjusted odds of DGF (OR 0.49, 95%CI 0.26-0.95). The only other risk factor independently associated with reduced adjusted odds of DGF was terminal donor serum creatinine (OR 1.66, 95%CI 1.19-2.31). There

							were no differences in the incidence of acute rejection, primary nonfunction, graft loss, or death were observed.
Wang et al. [28]	2017	Prospective, kidney pairs	Single Center, China	LifePort	UW	ECD, DBD, DCD	HMP decreased the incidence of DGF (16.7% vs. 37.5%, P=0.033) and mean length of hospital stay (12.3 vs. 19.4 days, P=0.001), but had no difference in acute rejection or survival compared to SCS among ECD grafts. Serum creatinine at discharge was improved in the HMP group (1.21 mg/dL vs. 1.37 mg/dL , P=0.004), but there was no difference at 6-month follow-up.
Zhong et al. [29]	2017	Prospective, kidney pairs	Single Center, China	LifePort	UW	DCD	HMP had lower rates of DGF (22.0% vs. 33.3%, P = 0.033) and functional-DGF (22.7% vs. 34.0%, P=0.035). The resistive indices within 48 hours after transplantation were reduced in the renal main artery, sub segmental artery, and interlobular artery (P<0.05) were lower in HMP. Patients with HMP grafts had higher post-transplant urine volume (4080 mL vs. 3000 mL, P = 0.047) and lower median serum creatinine (2.04 vs. 4.41 mg/dL, P=0.024) within 7 days. HMP had higher 1- (98% vs. 93%, P=0.026) and 3-year graft survival rates (93% vs. 82%, P=0.036).
Kox et al. [30]	2018	RCT, kidney pairs	Multicenter, Europe	LifePort	UW, HTK/Celsior, other	DBD, DCD	HMP grafts had reduced odds of DGF on multivariable analysis (OR 0.015, P=0.007). The effect of HMP was the greatest in the subgroup with the least amount of CIT (CIT<10 hours). CIT was an independent risk factor for DGF among HMP grafts after DBD (OR 1.06; 95%CI 1.02-1.12, P=0.008), DCD (OR 1.13; 95%CI, 1.04-1.23, P=0.006) and ECD (OR 1.14, 95%CI 1.057-1.236, P = 0.001).
Arlaban et al. [31]	2019	Prospective, kidney pairs	Single center, Spain	LifePort	N/A	cDCD, ECD	No difference in DGF or long-term graft function despite longer CIT in the HMP cohort of cDCD grafts (20.1 vs. 6.1 hours, P<0.001).
Basu et al. [32]	2019	Retrospective	Single center, USA	RM3 or LifePort	Belzer MP Solution	ECD	There were no differences between the two groups in DGF rate, and 6-year patient and graft survival despite longer CIT (14 vs. 11.2 hours,

							P<0.001) and higher terminal donor creatinine (1.3 vs 1.2 mg/dL, P=0.01). On multivariable logistic regression analysis, HMP reduced the risk of graft failure (hazard ratio [HR], 0.34; 95%CI 0.17-0.68) and death-censored graft failure (HR, 0.44; 95% CI, 0.19, 1.00), compared to CS subjects when DGF was not present.
Samoylova et al. [33]	2019	Retrospective	Multicenter, USA	Various	Various	DBD, DCD, ECD, SCD	MP kidneys came from older donors, DCD, and had longer CIT. MP treated kidneys had lower odds of DGF (OR 0.91; 95%CI 0.86-0.97) and rejection at 1 year (OR O.91; 95%CI 0.86-0.97). Data on 1-year graft loss was not used as it appears that there is an error in the reported tables regarding these data.
Foucher et al. [34]	2019	Retrospective	Multicenter, France	LifePort or Walters Medical Systems	N/A	ECD	Over 8 years of follow-up, recipients with grafts maintained by HMP had a mean life expectancy of 5.7 years (95%CI 5.4–6.1) vs 6.0 years (95% CI 5.7–6.2) in SCS group (P=0.155). Results were similar among patients receiving grafts from donors aged ≥70 years and in the transplantations with cold ischemia time ≥18 hours. HMP had higher donor age (69.4 vs. 68.2 years, P{<0.001), recipients who spent more time on the waitlist (26.5 vs. 26.4 months, P=0.005), but shorter CIT (5.7 vs. 18.2 hours, P<0.001).
Meister et al. [35]	2020	RCT	Single center, Germany	Kidney Transport Assist	HTK/Celsior	DBD, ECD	Tested hypothermic oxygenated machine perfusion (HOPE) on ECD grafts. No difference in in DGF, but patients after SCS had higher 6-month graft survival (100% vs. 87%, p=0.041). SCS had improved graft functioning: lower serum creatinine and higher eGFR rates on postoperative days 7 and 14. The decrease in renal vascular resistance was greatest in grafts with immediate function compared to those with DGF (p = 0.013).

Summers et al. [36]	2020	RCT, kidney pairs	Multicenter, UK	LifePort	KPS-1, UW, Marshall's, other	DCD	No difference in the incidence of DGF, PNF, hospital length of stay. The only difference observed was a higher eGFR at 3 months in HMP (44.8 vs. 33.6 mL/min, P=0.006), but there was no difference at 1 year follow-up. The trial stopped early due to difficulty with recruitment and the analysis was underpowered.
Husen et al. [37]	2021	RCT	Multicenter, Europe	Organ Assist BV	UW	DCD, ECD	A comparison between SCS or oxygenated HMP (HMPo2). HMPo2 had higher overall survival (7.1% vs. 1.5%, P=0.03). There was no difference in 1-year graft survival, DGF, or PNF. Because of the high overall graft survival rate (92.1% HMPo2 vs. 93.3% SCS, P=0.71), the study is underpowered.
Kruszyna et al. [38]	2021	Retrospective	Single center, Poland	LifePort	HTK/Celsior	DBD, ECD and SCD	HMP had higher CIT (24 vs 20 hours, P<0.05), but still reduced the rate of DGF (21.8% vs 42.6%, P<0.05). On multivariable logistic regression analysis, HMP had a 60% decreased odds of DGF (OR 0.4, 95%CI 0.2-0.7, P<0.002). Recipient male sex was also an independent predictor (OR 0.6, 95%CI 0.4-0.9) of DGF. The benefits of HMP over SCS was also redemonstrated when analyzing only paired kidneys (OR0.3, 95%CI 0.12-0.6, P=0.002). There were no differences in overall 1- and 5-year survival rates.
Weberskirch et al. [39]	2022	Prospective	Single center, Germany	LifePort	UW, HTK/Celsior	ECD, DBD, DCD	The incidence of DGF, serum creatinine at discharge and at 1-year post-transplant were similar, though CIT (16.5 vs. 11.3 hours, P<0.001) and history of dialysis (84.6 vs. 76.7 months, P=0.031) were longer in HMP. Flow rate and organ resistance at the start of HMP can be predictive of graft function as they were significantly worse in grafts that eventually suffered from DGF (lower arterial flow, P=0.026; higher organ resistance, P=0.005). Donor male sex was the only independent predictor of DGF in the multivariable logistic regression analysis (OR 2.22, 95%CI 1.02-4.87).

Malinoski et al. [40]	2023	RCT	Multicenter, USA	N/A	N/A	DBD	Evaluated whether therapeutic hypothermic cooling prior to transplant alone was noninferior to HMP and if the combination of both methods yielded benefit. The aRR of DGF with hypothermia were 1.72 (vs. HMP; 95%CI 1.35-2.17), 1.57 (vs. hypothermia+HMP; 95%CI, 1.26-1.96). The aRR for hypothermia+HMP was 1.09 (vs. HMP; 95% CI, 0.85-1.40). 1-year graft survival were similar between all groups.
Hosgood et al. [41]	2023	RCT	Multicenter, UK	Medtronic	N/A	DCD	NMP were associated with improved graft function (eGFR) at 1 year follow-up and its benefits persisted up to 2 years. No differences in DGF, PNF, patient or graft survival rates.
Choudhary et al. [42]	2022	Prospective, Kidney Pairs	Single center, India	NMP: Kidney Assist	N/A	DBD	DCD maintained with NRP were compared with DBD with SCS. There were no differences in graft function, DGF, graft loss at 1-year.

Table S3. Summary of NRP vs. ISP studies.

Authors	Year	Study Design	Centers, Country	Donor Types Involved	Main Findings
Valero et al. [43]	2000	Prospective	Single center, Spain	DCD	Compared SCS, total body cooling, and NRP. NRP had lower incidences of DGF (12.5% NRP vs 55% SCS vs 75% TBC) and PNF (0% NRP vs. 22.5% SCS vs. 0% TBC) compared to SCS or total body cooling. Serum creatinine levels lower than 3 mg/dL were achieved in fewer days in the normothermic recirculation group than in the others (P=0.029)
Farney et al. [44]	2011	Retrospective	Single center, USA	DCD: SCD, ECD	NRP reduced the incidence of DGF (21% vs. 60%, P=0.002), though there were no difference in PNF, acute rejection, patient and graft survival (1- and 3-years follow-up). At 1-month follow-up, eGFR was greater in the NRP group (50 vs. 37, P=0.007), but this benefit disappears at 1-year follow-up. In a sub-analysis comparing NRP to local non-NRP grafts with CIT <30 hours, NRP grafts had lower renovascular resistance, eGFR at 1-month and hospital length of stay (P<0.05).
Miranda-Utera et al. [45]	2015	Retrospective	Single center, Spain	DCD, DBD	DCD maintained with NRP were compared with DBD with SCS. Over the follow-up period, there were no differences in PNF, graft or patient survival. Patients with NRP had higher rates of DGF (80.9% vs. 46.8%, {<0.001),
Miñambres et al. [46]	2017	Retrospective	Single center, Spain	cDCD, ECD	DCD maintained with NRP were compared with DBD with SCS. No differences in DGF, graft survival. Also looked at outcomes of the transplanted liver, lungs and pancreas.
Foss et al. [47]	2018	Prospective	Single center, Norway	cDCD, DBD	DCD maintained with NRP were compared with DBD with SCS. There were no differences in graft function, DGF, graft loss at 1-year.
Demiselle et al. [48]	2016	Prospective	Multicenter, France	uDCD, ECD, SCD	DCD maintained with NRP were compared with DBD with SCS. There were no differences in graft function, DGF, graft loss at 1-year.
Molina et al. [49]	2018	Prospective	Single center, Spain	uDCD, DBD	uDCD grafts were preserved with NRP, while DBD grafts were maintained with SCS. NRP grafts had higher incidence of DGF (73.4% vs. 46.4%, P<0.01) 5- and 10-year patient and graft survival were similar. SCS grafts had superior graft function (eGFR) over NRP until 6-months post-transplant.
Delsuc et al. [50]	2018	Retrospective	Single center, France	uDCD	There were no differences in incidence of PNF, DGF, interstitial fibrosis, or operative complications, but NRP was associated with less dialysis days (8 vs. 15 days, P=0.05) and hospital length of stay (17 vs. 24 days, P=0.003). NRP grafts had better graft function as measured by eGFR at 1-year post-transplant and continued up until 2-years.

del Río et al. [51]	2019	Retrospective	Multicenter, Spain	uDCD, DBD	Compared in-situ cooling, NRP, and HRP. Odds 1-year graft loss were increased by in situ cooling (OR 5.6, 95%CI 2.7-11.5) compared to NRP on Cox regression analysis. Donor age≥60 years (OR 2.7, 95%CI 1.2-6.1) and prior history of a transplant (OR 3.5, 95%CI 1.5-8.3) were other predictors of 1-year graft loss. There were no differences in outcomes between HRP and NRP.
Antoine et al. [52]	2020	Prospective	Multicenter, France	uDCD	SCS grafts had higher odds of PNF (OR 4.51, 95%CI 1.34-15.2) and poor renal function at 1 year (OR 2.6, 95% 1.45-4.55) on multivariable analysis. Higher BMI (OR 1.2, 95%CI 1.09-1.23) were also associated with poor renal function at 1 year while donor age was associated with PNF (OR 0.95, 95%CI 0.91-0.99).
Mori et al. [53]	2020	Prospective	Single center, Italy	cDCD, DBD	cDCD grafts were maintained with NRP, DBD with SCS. There were no differences in DGF, PNF, graft or patient survival.
Padilla et al. [54]	2021	Retrospective	Multicenter, Spain	DCD	NRP had lower incidences of DGF (30.3% vs. 48.4%, P<0.001), lower serum creatinine at 1 year (1.5 vs. 1.8 mg/dL, P<0.001), and higher patient survival at 1 year (97.6% vs. 95.6%, P=0.015). After propensity score matched regression analyses, there were no differences in PNF or 1-year patient mortality. However, NRP was associated with lower odds of DGF (OR 1.97, 95%CI 1.43-2.72) and 1-year graft loss (HR 1.77, 95%CI 1.01-3.17).
Pearson et al. [55]	2021	Prospective	Single center, UK	DCD, DBD	Compared NRP to DCD and DBD under SCS. NRP, DCD, and DBD had comparable rates of DGF. However, after propensity score matching and multivariate regression, NRP was a significant covariate and grafts treated with NRP had lower rates of DGF (P=0.03). Transplant type was also an independent predictor of eGFR on multivariate analysis at 7, 14, and 30 days (P<0.05). Although significant the effect sizes decreased over time and authors postulated that it may be explained by the recovery from IRI.

REFERENCES

1. Alijani, M.; Cutler, J.; DelValle, C.; Morres, D.; Fawzy, A.; Pechan, B.; Helfrich, G. Single-Donor Cold Storage versus Machine Perfusion in Cadaver Kidney Preservation. **1985**, *40*, doi:10.1097/00007890-198512000-00017.
2. Merion, R.; Oh, H.; Port, F.; Toledo-Pereyra, L.; Turcotte, J. A Prospective Controlled Trial of Cold-Storage versus Machine-Perfusion Preservation in Cadaveric Renal Transplantation. **1990**, *50*.
3. Matsuno, N.; Sakurai, E.; Tamaki, I.; Uchiyama, M.; Kozaki, K.; Kozaki, M. The Effect of Machine Perfusion Preservation versus Cold Storage on the Function of Kidneys from Non-Heart-Beating Donors. **1994**, *57*.
4. Daemen, J.H.C.; De Vries, B.; Kootstra, G. The Effect of Machine Perfusion Preservation on Early Function of Non-Heart-Beating Donor Kidneys. *Transplantation Proceedings* **1997**, *29*, 3489, doi:10.1016/S0041-1345(97)00991-3.
5. Kosieradzki, M.; Danielewicz, R.; Kwiatkowski, A.; Polak, W.; Węgrowicz-Rebandel, I.; Wałaszewski, J.; Gaciong, Z.; Lao, M.; Rowiński, W. Rejection Rate and Incidence of Acute Tubular Necrosis after Pulsatile Perfusion Preservation. *Transplantation Proceedings* **1999**, *31*, 278–279, doi:10.1016/S0041-1345(98)01626-1.
6. Sellers, M.T.; Gallichio, M.H.; Hudson, S.L.; Young, C.J.; Bynon, J.S.; Eckhoff, D.E.; Deierhoi, M.H.; Diethelm, A.G.; Thompson, J.A. Improved Outcomes in Cadaveric Renal Allografts with Pulsatile Preservation. *Clinical Transplantation* **2000**, *14*, 543–549, doi:10.1034/j.1399-0012.2000.140605.x.
7. Kwiatkowski, A.; Wszola, M.; Kosieradzki, M.; Danielewicz, R.; Ostrowski, K.; Domagala, P.; Lisik, W.; Nosek, R.; Fesolowicz, S.; Trzebicki, J.; et al. Machine Perfusion Preservation Improves Renal Allograft Survival. *American Journal of Transplantation* **2007**, *7*, 1942–1947, doi:10.1111/j.1600-6143.2007.01877.x.
8. Plata-Munoz, J.J.; Muthusamy, A.; Quiroga, I.; Contractor, H.H.; Sinha, S.; Vaidya, A.; Darby, C.; Fuggle, S.V.; Friend, P.J. Impact of Pulsatile Perfusion on Postoperative Outcome of Kidneys from Controlled Donors after Cardiac Death. *Transplant International* **2008**, *21*, 899–907, doi:10.1111/j.1432-2277.2008.00685.x.
9. Reznik, O.N.; Bagnenko, S.F.; Loginov, I.V.; Iljina, V.A.; Ananyev, A.N.; Eremich, S.V.; Moysyuk, Y.G. Machine Perfusion as a Tool to Select Kidneys Recovered From Uncontrolled Donors After Cardiac Death. *Transplantation Proceedings* **2008**, *40*, 1023–1026, doi:10.1016/j.transproceed.2008.03.052.
10. Shah, A.P.; Milgrom, D.P.; Mangus, R.S.; Powelson, J.A.; Goggins, W.C.; Milgrom, M.L. Comparison of Pulsatile Perfusion and Cold Storage for Paired Kidney Allografts. *Transplantation* **2008**, *86*, 1006–1009, doi:10.1097/TP.0b013e318187b978.
11. Kwiatkowski, A.; Wszoła, M.; Kosieradzki, M.; Danielewicz, R.; Ostrowski, K.; Domagała, P.; Lisik, W.; Fesołowicz, S.; Michalak, G.; Trzebicki, J.; et al. The Early and Long Term Function and Survival of Kidney Alografts Stored before Transplantation by Hypothermic Pulsatile Perfusion. A Prospective Randomized Study. *Ann Transplant* **2009**, *14*, 14–17.

12. Jochmans, I.; Moers, C.; Smits, J.M.; Leuvenink, H.G.D.; Treckmann, J.; Paul, A.; Rahmel, A.; Squifflet, J.-P.; Van Heurn, E.; Monbaliu, D.; et al. Machine Perfusion Versus Cold Storage for the Preservation of Kidneys Donated After Cardiac Death: A Multicenter, Randomized, Controlled Trial. *Annals of Surgery* **2010**, *252*, 756–764, doi:10.1097/SLA.0b013e3181ffc256.
13. Watson, C.J.E.; Wells, A.C.; Roberts, R.J.; Akoh, J.A.; Friend, P.J.; Akyol, M.; Calder, F.R.; Allen, J.E.; Jones, M.N.; Collett, D.; et al. Cold Machine Perfusion Versus Static Cold Storage of Kidneys Donated After Cardiac Death: A UK Multicenter Randomized Controlled Trial. *American Journal of Transplantation* **2010**, *10*, 1991–1999, doi:10.1111/j.1600-6143.2010.03165.x.
14. Abboud, I.; Antoine, C.; Gaudez, F.; Fieux, F.; Lefaucheur, C.; Pillebout, E.; Viglietti, D.; Serrato, T.; Vérine, J.; Flamant, M.; et al. Pulsatile Perfusion Preservation for Expanded-Criteria Donors Kidneys: Impact on Delayed Graft Function Rate. *Int J Artif Organs* **2011**, *34*, 513–518, doi:10.5301/IJAO.2011.8458.
15. Treckmann, J.; Moers, C.; Smits, J.M.; Gallinat, A.; Maathuis, M.-H.J.; Van Kasterop-Kutz, M.; Jochmans, I.; Homan Van Der Heide, J.J.; Squifflet, J.-P.; Van Heurn, E.; et al. Machine Perfusion versus Cold Storage for Preservation of Kidneys from Expanded Criteria Donors after Brain Death: Machine Perfusion for ECD Kidneys. *Transplant International* **2011**, *24*, 548–554, doi:10.1111/j.1432-2277.2011.01232.x.
16. Gallinat, A.; Moers, C.; Treckmann, J.; Smits, J.M.; Leuvenink, H.G.D.; Lefering, R.; Van Heurn, E.; Kirste, G.R.; Squifflet, J.-P.; Rahmel, A.; et al. Machine Perfusion versus Cold Storage for the Preservation of Kidneys from Donors >=65 Years Allocated in the Eurotransplant Senior Programme. *Nephrology Dialysis Transplantation* **2012**, *27*, 4458–4463, doi:10.1093/ndt/gfs321.
17. Hanf, W.; Codas, R.; Meas-Yedid, V.; Berthiller, J.; Buron, F.; Chauvet, C.; Brunet, M.; Giroud, A.; McGregor, B.C.; Olivo-Marin, J.C.; et al. Kidney Graft Outcome and Quality (After Transplantation) From Uncontrolled Deceased Donors After Cardiac Arrest. *American Journal of Transplantation* **2012**, *12*, 1541–1550, doi:10.1111/j.1600-6143.2011.03983.x.
18. Moers, C.; Pirenne, J.; Paul, A.; Ploeg, R. Machine Perfusion or Cold Storage in Deceased-Donor Kidney Transplantation. **2012**, *366*, doi:10.1056/NEJMc1111038.
19. Cannon, R.M.; Brock, G.N.; Garrison, N.R.; Smith, J.W.; Marvin, M.R.; Franklin, G.A. To Pump or Not to Pump: A Comparison of Machine Perfusion vs Cold Storage for Deceased Donor Kidney Transplantation. *Journal of the American College of Surgeons* **2013**, *216*, 625–633, doi:10.1016/j.jamcollsurg.2012.12.025.
20. Sedigh, A.; Tufveson, G.; Bäckman, L.; Biglarnia, A.-R.; Lorant, T. Initial Experience With Hypothermic Machine Perfusion of Kidneys From Deceased Donors in the Uppsala Region in Sweden. *Transplantation Proceedings* **2013**, *45*, 1168–1171, doi:10.1016/j.transproceed.2012.10.017.
21. Tozzi, M.; Franchin, M.; Soldini, G.; Ietto, G.; Chiappa, C.; Maritan, E.; Villa, F.; Carcano, G.; Dionigi, R. Impact of Static Cold Storage VS Hypothermic Machine Preservation on Ischemic Kidney Graft: Inflammatory Cytokines and Adhesion Molecules as Markers of Ischemia/Reperfusion Tissue Damage. Our Preliminary Results. *International Journal of Surgery* **2013**, *11*, S110–S114, doi:10.1016/S1743-9191(13)60029-1.

22. Dion, M.S.; McGregor, T.B.; McAlister, V.C.; Luke, P.P.; Sener, A. Hypothermic Machine Perfusion Improves D Oppler Ultrasonography Resistive Indices and Long-term Allograft Function after Renal Transplantation: A Single-centre Analysis. *BJU International* **2015**, *116*, 932–937, doi:10.1111/bju.12960.
23. Guy, A.; McGrogan, D.; Inston, N.; Ready, A. Hypothermic Machine Perfusion Permits Extended Cold Ischemia Times with Improved Early Graft Function. **2015**, *13*, doi:10.6002/ect.2014.0174.
24. Yao, L.; Zhou, H.; Wang, Y.; Wang, G.; Wang, W.; Chen, M.; Zhang, K.; Fu, Y. Hypothermic Machine Perfusion in DCD Kidney Transplantation: A Single Center Experience. *Urologia Internationalis* **2015**, *96*, 148–151, doi:10.1159/000431025.
25. Forde, J.C.; Shields, W.P.; Azhar, M.; Daly, P.J.; Zimmermann, J.A.; Smyth, G.P.; Eng, M.P.; Power, R.E.; Mohan, P.; Hickey, D.P.; et al. Single Centre Experience of Hypothermic Machine Perfusion of Kidneys from Extended Criteria Deceased Heart-Beating Donors: A Comparative Study. *Ir J Med Sci* **2016**, *185*, 121–125, doi:10.1007/s11845-014-1235-8.
26. Moser, M.A.J.; Ginther, N.; Luo, Y.; Beck, G.; Ginther, R.; Ewen, M.; Matsche-Neufeld, R.; Shoker, A.; Sawicki, G. Early Experience with Hypothermic Machine Perfusion of Living Donor Kidneys - a Retrospective Study. *Transpl Int* **2017**, *30*, 706–712, doi:10.1111/tri.12964.
27. Tedesco-Silva, H.; Mello Offerni, J.C.; Ayres Carneiro, V.; Ivani De Paula, M.; Neto, E.D.; Brambate Carvalhinho Lemos, F.; Requião Moura, L.R.; Pacheco E Silva Filho, A.; De Morais Cunha, M.D.F.; Francisco Da Silva, E.; et al. Randomized Trial of Machine Perfusion Versus Cold Storage in Recipients of Deceased Donor Kidney Transplants With High Incidence of Delayed Graft Function. *Transplantation Direct* **2017**, *3*, e155, doi:10.1097/TXD.0000000000000672.
28. Wang, W.; Xie, D.; Hu, X.; Yin, H.; Liu, H.; Zhang, X. Effect of Hypothermic Machine Perfusion on the Preservation of Kidneys Donated After Cardiac Death: A Single-Center, Randomized, Controlled Trial. *Artificial Organs* **2017**, *41*, 753–758, doi:10.1111/aor.12836.
29. Zhong, Z.; Lan, J.; Ye, S.; Liu, Z.; Fan, L.; Zhang, Y.; Fu, Z.; Qiao, B.; Shiu-Chung Ko, D.; Wang, Y.; et al. Outcome Improvement for Hypothermic Machine Perfusion Versus Cold Storage for Kidneys From Cardiac Death Donors. *Artificial Organs* **2017**, *41*, 647–653, doi:10.1111/aor.12828.
30. Kox, J.; Moers, C.; Monbaliu, D.; Strelniece, A.; Treckmann, J.; Jochmans, I.; Leuvenink, H.; Van Heurn, E.; Pirenne, J.; Paul, A.; et al. The Benefits of Hypothermic Machine Preservation and Short Cold Ischemia Times in Deceased Donor Kidneys. *Transplantation* **2018**, *102*, 1344–1350, doi:10.1097/TP.0000000000002188.
31. Arlaban, M.; Barreda, P.; Ballesteros, M.A.; Rodrigo, E.; Suberviola, B.; Valero, R.; Miñambres, E.; Ruiz-San Millán, J.C. Static Cold Storage vs Ex Vivo Machine Perfusion: Results From a Comparative Study on Renal Transplant Outcome in a Controlled Donation After Circulatory Death Program. *Transplantation Proceedings* **2019**, *51*, 311–313, doi:10.1016/j.transproceed.2018.12.010.

32. Basu, A.; Rosen, L.M.; Tan, H.P.; Fishbein, J.; Wu, C.M.; Donaldson, J.B.; Stuart, S.; `Shah, N.A.; McCauley, J.; Humar, A.; et al. Outcomes of Deceased Donor Kidney Transplantation Using Expanded Criteria Donor Kidneys Following Pulsatile Preservation. *Cureus* **2019**, doi:10.7759/cureus.5091.
33. Samoylova, M.L.; Nash, A.; Kuchibhatla, M.; Barbas, A.S.; Brennan, T.V. Machine Perfusion of Donor Kidneys May Reduce Graft Rejection. *Clinical Transplantation* **2019**, *33*, e13716, doi:10.1111/ctr.13716.
34. Foucher, Y.; Fournier, M.-C.; Legendre, C.; Morelon, E.; Buron, F.; Girerd, S.; Ladrière, M.; Mourad, G.; Garrigue, V.; Glotz, D.; et al. Comparison of Machine Perfusion versus Cold Storage in Kidney Transplant Recipients from Expanded Criteria Donors: A Cohort-Based Study. *Nephrology Dialysis Transplantation* **2020**, *35*, 1051–1059, doi:10.1093/ndt/gfz175.
35. Meister, F.A.; Czigany, Z.; Rietzler, K.; Miller, H.; Reichelt, S.; Liu, W.-J.; Boecker, J.; Moeller, M.J.; Tolba, R.H.; Hamesch, K.; et al. Decrease of Renal Resistance during Hypothermic Oxygenated Machine Perfusion Is Associated with Early Allograft Function in Extended Criteria Donation Kidney Transplantation. *Sci Rep* **2020**, *10*, 17726, doi:10.1038/s41598-020-74839-7.
36. Summers, D.M.; Ahmad, N.; Randle, L.V.; O'Sullivan, A.-M.; Johnson, R.J.; Collett, D.; Attia, M.; Clancy, M.; Tavakoli, A.; Akyol, M.; et al. Cold Pulsatile Machine Perfusion Versus Static Cold Storage for Kidneys Donated After Circulatory Death: A Multicenter Randomized Controlled Trial. *Transplantation* **2020**, *104*, 1019–1025, doi:10.1097/TP.0000000000002907.
37. Husen, P.; Boffa, C.; Jochmans, I.; Krikke, C.; Davies, L.; Mazilescu, L.; Brat, A.; Knight, S.; Wettstein, D.; Cseprekal, O.; et al. Oxygenated End-Hypothermic Machine Perfusion in Expanded Criteria Donor Kidney Transplant: A Randomized Clinical Trial. *JAMA Surg* **2021**, *156*, 517, doi:10.1001/jamasurg.2021.0949.
38. Kruszyna, T.; Richter, P. Hypothermic Machine Perfusion of Kidneys Compensates for Extended Storage Time: A Single Intervention With a Significant Impact. *Transplantation Proceedings* **2021**, *53*, 1085–1090, doi:10.1016/j.transproceed.2021.01.022.
39. Weberskirch, S.; Katou, S.; Reuter, S.; Kneifel, F.; Morgul, M.; Becker, F.; Houben, P.; Pascher, A.; Vogel, T.; Radunz, S. Dynamic Parameters of Hypothermic Machine Perfusion—An Image of Initial Graft Function in Adult Kidney Transplantation? *JCM* **2022**, *11*, 5698, doi:10.3390/jcm11195698.
40. Malinoski, D.; Saunders, C.; Swain, S.; Groat, T.; Wood, P.R.; Reese, J.; Nelson, R.; Prinz, J.; Kishish, K.; Van De Walker, C.; et al. Hypothermia or Machine Perfusion in Kidney Donors. *N Engl J Med* **2023**, *388*, 418–426, doi:10.1056/NEJMoa2118265.
41. Hosgood, S.; Callaghan, C.; Wilson, C.; Smith, L.; Mullings, J.; Mehew, J.; Oniscu, G.; Phillips, B.; Bates, L.; Nicholson, M. Normothermic Machine Perfusion versus Static Cold Storage in Donation after Circulatory Death Kidney Transplantation: A Randomized Controlled Trial. **2023**, *29*, doi:10.1038/s41591-023-02376-7.
42. Choudhary, D.; Sharma, A.; Singh, S.; Kenwar, D.B.; Walker Minz, R.; Singh Kohli, H.; Nada, R.; Wangkheimayum, S.; Jain, K.; Patil, S.S. Application of Ex Vivo Normothermic Machine Perfusion in Deceased Donors With Acute Kidney Injury With

Successful Renal Transplantation: A Preliminary Experience. *Transplantation Direct* **2022**, *8*, e1391, doi:10.1097/TXD.0000000000001391.
43. Valero, R.; Cabrer, C.; Oppenheimer, F.; Trias, E.; Sánchez-Ibáñez, J.; Cabo, F.M.D.; Navarro, A.; Paredes, D.; Alcaraz, A.; Gutiérrez, R.; et al. Normothermic Recirculation Reduces Primary Graft Dysfunction of Kidneys Obtained from Non-Heart-Beating Donors. *Transplant Int* **2000**, *13*, 303–310, doi:10.1111/j.1432-2277.2000.tb01086.x.
44. Farney, A.C.; Hines, M.H.; al-Geizawi, S.; Rogers, J.; Stratta, R.J. Lessons Learned from a Single Center's Experience with 134 Donation after Cardiac Death Donor Kidney Transplants. *Journal of the American College of Surgeons* **2011**, *212*, 440–451, doi:10.1016/j.jamcollsurg.2010.12.033.
45. Miranda-Utrera, N.; Medina-Polo, J.; Pamplona-Casamayor, M.; Passas-Martínez, J.B.; Rodríguez-Antolín, A.; De La Rosa Kehrmann, F.; Duarte-Ojeda, J.M.; Tejido-Sánchez, A.; Villacampa Aubá, F.; Andrés Belmonte, A. Uncontrolled Non-Heartbeating Donors (Types I–II) with Normothermic Recirculation vs. Heartbeating Donors: Evaluation of Functional Results and Survival. *Actas Urológicas Españolas (English Edition)* **2015**, *39*, 429–434, doi:10.1016/j.acuroe.2015.06.007.
46. Miñambres, E.; Suberviola, B.; Dominguez-Gil, B.; Rodrigo, E.; Ruiz-San Millan, J.C.; Rodríguez-San Juan, J.C.; Ballesteros, M.A. Improving the Outcomes of Organs Obtained From Controlled Donation After Circulatory Death Donors Using Abdominal Normothermic Regional Perfusion. *American Journal of Transplantation* **2017**, *17*, 2165–2172, doi:10.1111/ajt.14214.
47. Foss, S.; Nordheim, E.; Sørensen, D.W.; Syversen, T.B.; Midtvedt, K.; Åsberg, A.; Dahl, T.; Bakkan, P.A.; Foss, A.E.; Geiran, O.R.; et al. First Scandinavian Protocol for Controlled Donation After Circulatory Death Using Normothermic Regional Perfusion. *Transplantation Direct* **2018**, *4*, e366, doi:10.1097/TXD.0000000000000802.
48. Demiselle, J.; Augusto, J.-F.; Videcoq, M.; Legeard, E.; Dubé, L.; Templier, F.; Renaudin, K.; Sayegh, J.; Karam, G.; Blancho, G.; et al. Transplantation of Kidneys from Uncontrolled Donation after Circulatory Determination of Death: Comparison with Brain Death Donors with or without Extended Criteria and Impact of Normothermic Regional Perfusion. *Transpl Int* **2016**, *29*, 432–442, doi:10.1111/tri.12722.
49. Molina, M.; Guerrero-Ramos, F.; Fernández-Ruiz, M.; González, E.; Cabrera, J.; Morales, E.; Gutierrez, E.; Hernández, E.; Polanco, N.; Hernández, A.; et al. Kidney Transplant from Uncontrolled Donation after Circulatory Death Donors Maintained by nECMO Has Long-Term Outcomes Comparable to Standard Criteria Donation after Brain Death. *American Journal of Transplantation* **2019**, *19*, 434–447, doi:10.1111/ajt.14991.
50. Delsuc, C.; Faure, A.; Berthiller, J.; Dorez, D.; Matillon, X.; Meas-Yedid, V.; Floccard, B.; Marcotte, G.; Labeye, V.; Rabeyrin, M.; et al. Uncontrolled Donation after Circulatory Death: Comparison of Two Kidney Preservation Protocols on Graft Outcomes. *BMC Nephrol* **2018**, *19*, 3, doi:10.1186/s12882-017-0805-1.

51. Del Río, F.; Andrés, A.; Padilla, M.; Sánchez-Fructuoso, A.I.; Molina, M.; Ruiz, Á.; Pérez-Villares, J.M.; Peiró, L.Z.; Aldabó, T.; Sebastián, R.; et al. Kidney Transplantation from Donors after Uncontrolled Circulatory Death: The Spanish Experience. *Kidney International* **2019**, *95*, 420–428, doi:10.1016/j.kint.2018.09.014.
52. Antoine, C.; Savoye, E.; Gaudez, F.; Cheisson, G.; Badet, L.; Videcoq, M.; Legeai, C.; Bastien, O.; Barrou, B. Kidney Transplant From Uncontrolled Donation After Circulatory Death: Contribution of Normothermic Regional Perfusion. *Transplantation* **2020**, *104*, 130–136, doi:10.1097/TP.0000000000002753.
53. Mori, G.; Solazzo, A.; Tonelli, L.; Facchini, F.; Fontana, F.; Alfano, G.; Baroni, S.; Cappelli, G. Comparison Between Kidney Transplantation After Circulatory Death and After Brain Death: A Monocentric Retrospective Study After 1 Year of Follow-Up. *Transplantation Proceedings* **2020**, *52*, 1536–1538, doi:10.1016/j.transproceed.2020.02.043.
54. Padilla, M.; Coll, E.; Fernández-Pérez, C.; Pont, T.; Ruiz, Á.; Pérez-Redondo, M.; Oliver, E.; Atutxa, L.; Manciño, J.M.; Daga, D.; et al. Improved Short-Term Outcomes of Kidney Transplants in Controlled Donation after the Circulatory Determination of Death with the Use of Normothermic Regional Perfusion. *American Journal of Transplantation* **2021**, *21*, 3618–3628, doi:10.1111/ajt.16622.
55. Pearson, R.; Geddes, C.; Mark, P.; Clancy, M.; Asher, J. Transplantation of Kidneys after Normothermic Perfusion: A Single Center Experience. *Clinical Transplantation* **2021**, *35*, e14431, doi:10.1111/ctr.14431.
